# Lung function in adults born with very low birth weight from young to mid-adulthood

**DOI:** 10.1038/s41390-025-04246-z

**Published:** 2025-06-28

**Authors:** Laura Jussinniemi, Silje D. Benum, Kristina A. D. Aakvik, Maarit K. Kulmala, Anna P. M. Jørgensen, Sture Andersson, Petteri Hovi, Heli-Kaisa Leppänen, Sigurd Steinshamn, Kari Anne I. Evensen, Eero Kajantie

**Affiliations:** 1 Clinical Medicine Research Unit, MRC Oulu, Oulu University Hospital and University of Oulu, Oulu, Finland; 2 Welfare Epidemiology and Monitoring Unit, Finnish Institute for Health and Welfare, Helsinki, Finland; 3 Department of Clinical and Molecular Medicine, Norwegian University of Science and Technology, Trondheim, Norway; 4 Department of Ophthalmology, Helsinki University Hospital, University of Helsinki, Helsinki, Finland; 5 Department of Neuromedicine and Movement Science, Norwegian University of Science and Technology, Trondheim, Norway; 6 Department of Ophthalmology, St. Olavs Hospital, Trondheim University Hospital, Trondheim, Norway; 7 Children’s Hospital, Pediatric Research Center, University of Helsinki, Helsinki, Finland; 8 Helsinki University Hospital, Helsinki, Finland; 9 Department of Thoracic Medicine, St Olavs Hospital, Trondheim University Hospital, Trondheim, Norway; 10 Department of Circulation and Medical Imaging, Norwegian University of Science and Technology, Trondheim, Norway; 11 Department of Rehabilitation Science and Health Technology, Oslo Metropolitan University, Oslo, Norway; 12 Children’s Clinic, St. Olavs Hospital, Trondheim University Hospital, Trondheim, Norway

## Abstract

**Background:**

Young adults born preterm with very low birth weight (VLBW; <1500 g), especially those who had neonatal bronchopulmonary dysplasia (BPD), have reduced lung function compared with their term-born peers. We hypothesized that these impairments worsen between young and mid-adulthood.

**Methods:**

We studied two birth cohorts: HeSVA (Finland) and NTNU LBW Life (Norway) with harmonized protocols. Lung function was assessed by spirometry in 115 VLBW (20 with BPD) and 142 controls at mean 36 years. The results were compared with young adulthood (22 years) spirometry in 198 VLBW (32 with BPD) and 225 control participants.

**Results:**

In mid-adulthood, BPD-VLBW adults had lower z-scores in forced expiratory volume in 1 s (zFEV1; mean difference −1.49; 95%CI −1.94, −1.04) and in zFEV1/FVC (FVC: forced vital capacity; mean difference −0.84, 95%CI −1.23, −0.44) than controls. Corresponding differences for non-BPD-VLBW adults were −0.34 (95%CI −0.60, −0.08) and −0.30 (95%CI −0.53, −0.07). The differences in zFVC and zFEV1 between BPD-VLBW and control groups increased from young to mid-adulthood.

**Conclusion:**

In mid-adulthood, adults born preterm with VLBW had reduced airflow compared with term-born controls. These differences were more pronounced among those with a history of BPD, whose vital capacity also seems to decline faster.

**Impact:**

- Impaired airflow and reduced vital capacity in VLBW adults do not improve with age.
- Among those with a history of BPD, vital capacity seems to reduce faster than among their term-born peers.
- Clinicians who see adults born preterm with VLBW should be vigilant for respiratory symptoms. We argue that a full medical history in all adults who present with respiratory symptoms should routinely include key birth characteristics such as preterm birth.

## Introduction

Worldwide, on average 10% of infants are born preterm (<37 completed weeks of gestation)^1^ and ~1% with very low birth weight (VLBW < 1500 g).^2^

VLBW survivors are known to have poorer lung function in childhood^3–5^ and may not reach the full potential of airway growth in the early adulthood.^6,7^ Maximal expiratory airflow peaks in early adulthood and declines with age.^6,8^ Among those born preterm, there is emerging evidence of an increased risk of chronic obstructive pulmonary disease (COPD) in later adulthood^6,7,9^ and these risks are particularly large among those who had neonatal bronchopulmonary dysplasia (BPD),^7,10–12^ also known as chronic lung disease of prematurity. Children and young adults with a history of BPD have been suggested to have a more rapid decline in airflow over time.^13–17^ Accordingly, a history of BPD has been proposed as a risk factor for developing COPD in later life.^17,18^

The first generations of VLBW infants who received neonatal intensive care are now reaching middle age, but there is limited research on the rate of decline in lung function from young to mid-adulthood among those born preterm with VLBW with or without BPD.^19^

## Objectives

Our aim was to evaluate respiratory health of adults born preterm with VLBW with or without a history of BPD, as compared with their term-born counterparts in mid-adulthood and compare the mid-adulthood spirometry results with young adulthood spirometry results, which were now combined together from two cohorts and reanalyzed. We hypothesized that individuals born preterm with VLBW have impaired airflow compared with term-born controls and that the impairment is particularly strong among those who had neonatal BPD. In addition, we hypothesized that differences in airflow have increased since previous studies in young adulthood.

## Methods

### Study design

The data were collected with harmonized data collection and management procedures from two longitudinal birth cohorts; the Helsinki Study of Very Low Birth Weight Adults (HeSVA) in Helsinki, Finland and the NTNU Low Birth Weight in a Lifetime Perspective study (NTNU LBW Life) in Trondheim, Norway. The mid-adulthood follow-up visits were conducted in 2019–2021, including a respiratory health assessment by spirometry as part of an extensive health assessment.

The original HeSVA cohort has undergone detailed clinical assessment at ages 22 and 25 years. The cohort comprised 335 VLBW infants who were born between 1978 and 1985 and discharged alive from the neonatal intensive care unit of Helsinki University Central Hospital. For every VLBW infant was selected a term-born singleton infant of the same sex not born small for gestational age, group-matched for age, sex and birth hospital^20,21^. Lung function was examined in young adulthood during 2004–2005 by spirometry (Medikro) from 322 participants at a mean age of 22 years.^7^

The original NTNU LBW Life cohort have undergone detailed clinical assessment at ages 1, 5, 14, 18, 20, 23, and 26 years. The cohort comprised 88 VLBW infants who were born between 1986–1988 and discharged alive from the neonatal intensive care unit of St. Olavs Hospital, Trondheim, Norway. Control participants were recruited from a population-based multicenter study on causes and consequences of intrauterine growth restriction, where a 10% random sample was selected for follow-up during their mothers’ second or third pregnancy. Control participants were born at term and not small for gestational age in Trondheim region.^22^ Lung function was examined in young adulthood by spirometry (Sensormedics Vmax22 Encore) from 100 participants at mean age of 18 years.^23^

**Fig. 1:**
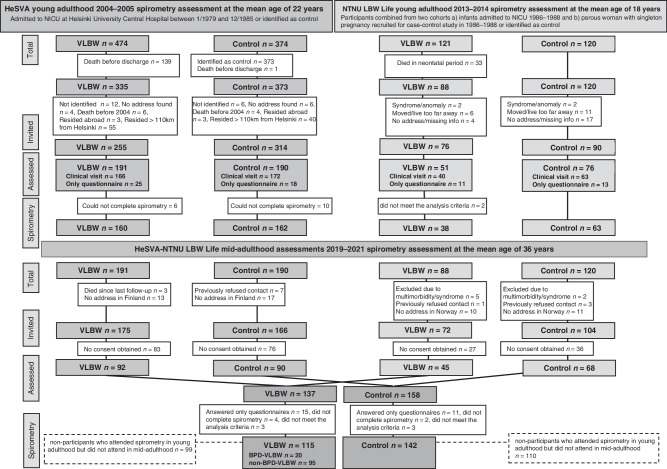
Flow chart from the HeSVA-NTNU LBW life participants at birth and in young and mid-adulthood assessments. BPD bronchopulmonary dysplasia, HeSVA Helsinki study of very low birth weight adults, NICU neonatal intensive care unit, NTNU LBW Life NTNU low birth weight in a lifetime perspective study, VLBW very low birth weight.

We combined and reanalyzed the young adulthood data from the HeSVA and NTNU LBW Life cohorts. The pooled young adulthood HeSVA-NTNU LBW Life dataset included altogether 423 participants at mean age 21.5 (SD 2.6) years; 110 VLBW women, 88 VLBW men, 129 control women and 96 control men (Fig. 1).

### Participants

The flow of study participants is described in Fig. 1.

A total of 175 VLBW adults from HeSVA and 72 from NTNU LBW Life were invited for mid-adulthood assessment at a mean age of 36 (SD 3.3) years and 137 participated (55.5% women). Of these, 15 answered questionnaires only and did not attend the clinical visits. Four participants were not able to complete spirometry due to technical reasons or having respiratory symptoms within the past two weeks before the clinical visit, and for three participants, spirometry values did not meet the reproducibility criteria.

In the spirometry analyses, we included 115 VLBW participants (59.1% women). Of these, 20 (60% women) had neonatal BPD and were included in the BPD-VLBW group, and 95 (58.9% women) participants without BPD were included in the non-BPD-VLBW group.

In our primary analyses, BPD was defined based on clinician’s diagnosis. In HeSVA, this was determined using the Northway criteria^24^ (respiratory distress, supplemental oxygen and characteristic radiographic findings at 1 month of postnatal age), all diagnoses confirmed by the same neonatologist, which has been used in our previous publications in HeSVA.^7,25^ For consistency, in NTNU, we used a clinician’s diagnosis collected from medical records. We also conducted sensitivity analyses using BPD definitions by the need for supplemental oxygen for more than 28 days (29 VLBW participants) and for more than 36 weeks (9 VLBW participants).

In the control group, 166 from HeSVA and 104 from NTNU LBW Life were invited and 158 (58.5% women) of them participated. Eleven control participants answered only questionnaires and did not attend clinical visits. Two control participants did not complete spirometry due to a shortage of staff. In the spirometry analyses, we included 142 control participants (57.7% women). Written informed consent was obtained from all participants who attended the study. The study protocols were approved by the ethics committee at the Helsinki and Uusimaa Hospital district (HUS/1157) and by the Regional Committee for Medical and Health Research Ethics in Central-Norway (23879). The protocol is registered as ISRCTN77533991.

### Measures

In mid-adulthood, lung function was measured by spirometry (Medikro in HeSVA and Sensormedics in NTNU LBW Life). Spirometry was performed in accordance with the American Thoracic Society/European Respiratory Society recommendations.^26^ In HeSVA, the participants were instructed not to take their medication in the morning of the examination. In NTNU LBW Life, they were asked to continue their medication, including anti-obstructive medication, as usual. Reversibility tests were not performed. The devices were calibrated daily before measurements. Spirometry was not performed if the participant had had a respiratory infection in the past two weeks prior to the clinical visit. Spirometry was performed with participants in a sitting position wearing nose clips and holding mouthpiece tightly between teeth and lips. Respiratory volumes were standardized to barometric pressure at sea level and body temperature. The maneuver was repeated until three technically acceptable spirometry curves were obtained. The largest value of forced vital capacity (FVC) and forced expiratory volume in 1 s (FEV1) were reported; the other measurements came from the maneuver where the sum of FVC and FEV1 was largest.^26,27^

Primary outcomes were FEV1 and ratio of the FEV1 to FVC (FEV1/FVC). Secondary outcomes were forced expiratory flow when 75% of FVC has been exhaled (FEF_75%_) and forced expiratory flow at 25–75% (FEF_25–75%_). All absolute values were converted to z-scores based on the Global Lung Function Initiative guidelines, which account for participant age, height, sex and race.^28^ Six participants were excluded from the analysis because they did not have (all) values for FEV1, FVC, FEV1/FVC or the values did not meet the reproducibility criteria according to American Thoracic Society and European Respiratory Society; for FEV1 and FVC the best two values had to be within 5% or 150 ml and for FEV1/FVC the difference of the sum of FEV1 and FVC had to be less than 300 ml. In addition, the participants answered the questionnaire on respiratory symptoms of the European Community Respiratory Health Survey.^29^ They were also asked to report the history of physician-diagnosed asthma and any regular or seasonal use of obstructive airway medication (ATC codes R03AC, R03AK, R03BA, R03DC).

### Statistical methods and power calculation

The data were analyzed with IBM SPSS Statistics, version 29.0.0.0 (241). A priori power calculation was based on a total population of 170 VLBW participants and 200 controls. With a statistical power of 80% and an alpha level of 0.05, the detectable difference in a continuous outcome between groups was 0.29 SD score and with 90% power and an alpha of 0.01 the detectable difference was 0.40 SD. Before data analysis, with the actual number of 137 VLBW and 158 controls participants, the corresponding numbers were 0.33 and 0.45 SD.^30^

We assessed normality and distributions by evaluating histograms and Q–Q Plot residuals. The baseline characteristics comparisons between BPD-VLBW or non-BPD-VLBW groups and term-born controls were done by t-tests and X^2^ tests. To detect whether the associations between VLBW birth and lung function differed by sex, we added an interaction term group*sex for zFVC, zFEV1 and zFEV1/FVC both in young and mid-adulthood. We only found significant group*sex interaction in mid-adulthood zFVC. As FEV1 and FEV1/FVC absolute and z-score variables served as our primary outcomes, we decided to report the results for women and men together.

The most important modifier of the associations between VLBW birth and lung function and respiratory health was a history of BPD. Therefore, we present the results with lung function as an outcome, comparing the BPD-VLBW and non-BPD-VLBW groups with controls by linear regression models separately in young and mid-adulthood. All linear regression analyses were adjusted for cohort, age and sex (model 1). In further adjustments, model 2 included, in addition highest parental educational attainment and model 3 highest parental educational attainment and maternal smoking. Model 3 was conducted only for HeSVA participants because NTNU LBW Life did not have data on maternal smoking in pregnancy. We used mixed model analysis to estimate whether the BPD-VLBW or non-BPD-VLBW participants would have a faster rate of decline in spirometry outcomes between young and mid-adulthood, as compared with controls. To do so, we added an interaction term “group (BPD-VLBW or non-BPD-VLBW vs. control) * assessment timepoint (young vs. mid-adulthood)” in the model that also included the main effects of the group and assessment timepoint, as well as cohort, sex and age at young adulthood assessment.

In addition, using the same methods, we conducted an analysis between BPD-VLBW and non-BPD-VLBW participants to assess differences within the VLBW group, adjusting for model 1 variables. As a further sensitivity analysis, we compared BPD-VLBW population spirometry outcomes with controls by defining BPD by the need for supplemental oxygen for more than 28 days or for more than 36 (postmenstrual) weeks.

To compare dichotomous outcomes, including respiratory symptoms or a history of obstructive airway disease, between the VLBW and control groups, we used logistic regression.

To estimate possible effects of the participant’s smoking on respiratory health we performed linear regression (adjustment model 1) in each time point within and between the VLBW and control participants who had reported their smoking status in the mid-adulthood visit questionnaire and had spirometry data available from both assessment points (*n* = 209) allowing age interaction to be assessed by mixed models. We conducted a dichotomous variable indicating whether the participant had any kind of smoking history compared with those who had never smoked. We conducted the comparisons between VLBW and control participants with smoking history and within the VLBW and control groups with or without smoking history.

## Results

### Background characteristics of the mid-adulthood visits

Main background and maternal characteristics are described in Table 1. VLBW participants had lower educational attainment (*p* = 0.049) and they reported more use of inhaled glucocorticoids or adrenergic bronchodilators (*p* = 0.02) compared with controls.

**Table 1:** Background characteristics from the mid-adulthood spirometry assessment.

	1. BPD-VLBW	2. Non-BPD-VLBW	3. (all) VLBW	4. Term (reference)	*p*-value between 1 and 4	*p*-value between 2 and 4	*p*-value between 3 and 4
**Number of participants**	20	95	115	142			
HeSVA	16	61	77	83			
NTNU	4	34	38	59			
**Maternal background characteristics**	**Mean (SD) or** ***n*** **(%)**	**Mean (SD) or** ***n*** **(%)**	**Mean (SD) or** ***n*** **(%)**	**Mean (SD) or** ***n*** **(%)**			
Maternal age (years)	29.5 (5.5)	30.2 (4.5)	30.1 (4.7)	30.05 (4.8)	0.66	0.84	0.98
Height (cm)	163.9 (7.3)	165.2 (6.0)	165.0 (6.2)	166.1 (5.6)	0.15	0.27	0.16
Smoking during pregnancy^a^	2 (20.0%)	12 (26.1%)	14 (25.0)	11 (16.9)	0.81	0.24	0.27
Parental educational attainment					0.73	0.11	0.12
Basic or less	4 (21.1%)	19 (20.4%)	23 (20.5%)	15 (11.5%)			
Upper secondary	4 (21.1%)	15 (16.1%)	19 (17.0%)	28 (21.4%)			
Lower-level tertiary	5 (26.3%)	32 (34.4%)	37 (33.0%)	37 (28.2%)			
Upper-level tertiary	6 (31.6%)	27 (29.0%)	33 (29.5%)	51 (38.9%)			
**Study participant characteristics**							
Sex, women	12 (60.0%)	56 (58.9%)	68 (59.1%)	82 (57.7%)	0.84	0.85	0.82
Gestational age (weeks)	27.5 (2.5)	29.8 (2.3)	29.4 (2.5)	40.1 (1.2)	<0.001	<0.001	<0.001
Birth weight (g)	997 (261)	1207 (193)	1171 (220)	3653 (481)	<0.001	<0.001	<0.001
Birth weight SD score Finnish reference	−0.87 (1.20)	−1.33 (1.69)	−1.24 (1.6)	0.14 (1.0)	0.001	<0.001	<0.001
Birth weight SD score Norwegian reference	−0.67 (0.90)	−1.09 (1.17)	−1.02 (1.1)	0.05 (1.0)	0.002	<0.001	<0.001
C-section as a delivery mode	8 (40.0%)	66 (70.2%)	74 (64.9%)	11 (13.3%)	0.010	<0.001	<0.001
Ventilator treatment (days)	32.3 (21.5)	3.3 (4.4)	8.2 (14.5)	-	-	-	-
Supplemental oxygen (days)	72.7 (84.3)	11.8 (15.3)	21.9 (42.8)	-	-	-	-
Age at discharge from hospital (days)	105.9 (82.2)	61.7 (20.5)	70.7 (44.2)	-	-	-	-
Bronchopulmonary dysplasia diagnosed by clinician	20 (100%)	-	20 (25.0%)	-	-	-	-
RDS	18% (90.0%)	37 (39.8%)	55 (48.7%)	-	-	-	-
**Study participant mid-adulthood assessment characteristics**							
Age (years)	36.3 (2.7)	36.1 (3.4)	36.1 (3.3)	35.8 (3.3)	0.48	0.24	0.36
Height, cm	164.0 (9.9)	168.9 (9.5)	168.0 (9.7)	173.7 (9.6)	<0.001	<0.001	<0.001
BMI (kg/m^2^)	27.4 (6.9)	25.7 (5.66)	26.0 (5.89)	25.7 (4.41)	0.29	0.91	0.58
Daily smoking	0 (0)	13 (14.0%)	13 (11.5%)	15 (10.6%)	0.04	0.43	0.81
Never smoked vs. others	14 (70.0%)	51 (54.8%)	65 (57.5%)	71 (50.0%)	0.09	0.47	0.23
Current use of inhaled glucocorticoids or adrenergic bronchodilators	4 (20.0%)	17 (17.9%)	21 (12.8%)	12 (8.5%)	0.12	0.03	0.02
History of asthma (treated or diagnosed by a physician)	4 (20.0%)	27 (28.4%)	31 (27.0%)	30 (21.1%)	1.0	0.20	0.28
Respiratory symptoms in the last 12 months	8 (40.0%)	32 (34.4%)	40 (35.4%)	44 (31.0%)	0.42	0.67	0.46
Educational attainment					0.14	0.003	0.049
Lower (ISCED levels 1–2)	0 (0)	3 (3.2%)	3 (2.6%)	2 (1.4%)			
Intermediate (ISCED levels 3–5)	11 (55%)	43 (45.3%)	54 (47.0%)	47 (33.1%)			
Lower tertiary or higher (ISCED levels 6–8)	9 (45.0%)	49 (51.6%)	58 (50.4%)	93 (65.5%)			

*BPD* bronchopulmonary dysplasia, *BMI* body mass index, *HeSVA* Helsinki study of very low birth weight adults, *ISCED* International Standard of Classification of Education, *NTNU* NTNU low birth weight in a lifetime perspective study, *SD* standard deviation, *VLBW* very low birth weight.

^a^Data available only for HeSVA participants.

### Lung function in mid-adulthood

The lung function results from BPD-VLBW or non-BPD-VLBW participants compared with controls are described in Table 2 and results from all VLBW participants in one group (with or without BPD) compared with controls are described in the Supplementary Table S1.

**Table 2:** Comparisons of lung function from young to mid-adulthood between BPD-VLBW and control participants and between non-BPD-VLBW and control participants.

	1. BPD-VLBW	2. non-BPD-VLBW	3. Term (reference)	BPD-VLBW vs. term		non-BPD-VLBW vs. term	
Young adulthood study (*n*)	32	166	225				
Age (years)	21.4 (2.5)	21.6 (2.5)	21.4 (2.6)				
Mid-adulthood study (*n*)	20	95	142				
Age (years)	36.3 (3.2)	36.1 (3.4)	35.8 (3.3)				
**Absolute values**	**Mean (SD)**	**Mean (SD)**	**Mean (SD)**	**Mean difference (95% CI)**^**a**^	***p*-value for age interaction**^**b**^	**Mean difference (95% CI)**^**a**^	***p*-value for age interaction**
FVC young adulthood (l)	3.86 (0.88)	4.34 (1.03)	4.62 (1.01)	−0.67 (−0.92 to −0.42)		−0.31 (−0.45 to −0.17)	
FVC mid-adulthood (l)	3.59(1.01)	4.37 (1.07)	4.76 (1.04)	−1.01 (−1.30 to −0.73)	0.005	−0.34 (−0.50 to −0.18)	0.63
FEV1 young adulthood (l)	3.16 (0.86)	3.72 (0.80)	4.08 (0.85)	−0.90 (−1.12 to −0.69)		−0.40 (−0.52 to −0.29)	
FEV1 mid-adulthood (l)	2.58 (0.82)	3.28 (0.77)	3.68 (0.76)	−0.99 (−1.22 to −0.75)	0.11	−0.36 (−0.49 to −0.22)	0.97
FEV1/FVC young adulthood (l)	81.49 (10.76)	86.64 (8.76)	88.97 (7.0)	−8.76 (−11.49 to −6.04)		−2.68 (−4.14 to −1.22)	
FEV1/FEVC mid-adulthood (%)	71.95 (8.70)	75.89 (7.60)	77.80 (5.51)	−5.96 (−8.77 to −3.15)	0.51	−1.93 (−3.52 to −0.34)	0.95
FEF_25–75%_ young adulthood^c^	3.40 (1.42)	4.33 (1.29)	4.85 (1.26)	−1.49 (−1.96 to −1.01)		−0.52 (−0.79 to −0.25)	
FEF_25–75%_ mid-adulthood	2.01 (0.88)	2.82 (0.89)	3.32 (0.83)	−1.22 (−1.58 to −0.85)	0.69	−0.48 (−0.68 to −0.27)	0.95
FEF_75%_ young adulthood^c^	1.83 (0.90)	2.49 (0.92)	2.80 (0.88)	−1.00 (−1.35 to −0.67)		−0.32 (−0.52 to −0.11)	
FEF_75%_ mid-adulthood	0.76 (0.37)	1.26 (0.51)	1.53 (0.50)	−0.70 (−0.91 to −0.48)	0.17	−0.25 (−0.37 to −0.13)	0.60
**z-scores**							
FVC young adulthood	−0.75 (1.07)	−0.31 (1.20)	−0.25 (1.04)	−0.41 (−0.81 to −0.02)		−0.04 (−0.27 to 0.18)	
FVC mid-adulthood	−1.10 (1.14)	−0.08 (1.15)	0.09 (0.91)	−1.04 (−1.48 to −0.61)	0.001	−0.12 (−0.38 to 0.13)	0.56
FEV1 young adulthood	−1.17 (1.36)	−0.27 (1.32)	0.04 (1.11)	−1.23 (−1.67 to −0.80)		−0.34 (−0.58 to −0.10)	
FEV1 mid-adulthood	−1.94 (1.26)	−0.70 (1.19)	−0.33 (0.91)	−1.49 (−1.94 to −1.04)	0.025	−0.34 −0.60 to −0.08)	0.64
FEV1/FVC young adulthood	-0.66 (1.48)	0.13 (1.37)	0.49 (1.16)	−1.35 (−1.78 to −0.93)		−0.44 (−0.68 to −0.21)	
FEV1/FEVC mid-adulthood	−1.52 (1.03)	−0.99 (1.03)	−0.70 (0.80)	−0.84 (−1.23 to −0.44)	0.34	−0.30 (−0.53 to −0.07)	0.91
FEF_25–75%_ young adulthood^c^	−1.02 (1.45)	−0.01 (1.26)	0.33 (1.10)	−1.32 (−1.79 to −0.86)		−0.33 (−0.60 to −0.06)	
FEF_25–75%_ mid-adulthood	−2.01 (1.28)	−1.05 (0.98)	−0.63 (0.81)	−1.34 (−1.76 to −0.92)	0.57	−0.41 (−0.64 to −0.19)	0.39
FEF_75%_ young adulthood^c^	−0.49 (1.20)	0.47 (1.11)	0.69 (0.97)	−1.15 (−1.56 to −0.75)		−0.22 (−0.46 to 0.02)	
FEF_75%_ mid-adulthood	−1.64 (1.22)	−0.55 (0.91)	−0.18 (0.76)	−1.41 (−1.81 to −1.01)	0.45	−0.36 (−0.57 to −0.15)	0.22

*BPD* bronchopulmonary dysplasia, *CI* confidence interval, *FVC* forced vital capacity, *FEV1* forced expiratory volume in 1s, *FEV1/FVC* ratio of the forced expiratory volume in the first 1s to the forced vital capacity, *FEF*_*25–75%*_, forced expiratory flow at 25–75%, *FEF*_*75%*_, forced expiratory flow when 75% of FVC has been exhaled, *VLBW* Very low birth weight, *SD* standard deviation.

^a^Linear regressions adjusted for cohort, age and sex.

^b^*p*-value for age interaction by mixed models.

^c^Data available only for Helsinki Study of Very Low Birth Weight adults (HeSVA) participants.

In mid-adulthood, adjusted for cohort, age and sex, BPD-VLBW participants had significantly lower values in all main outcomes (FVC, FEV1, FEV1/FVC, FEF_25–75%_ and FEF_75%_) in both absolute values and z-scores, compared with term-born controls. BPD-VLBW participants had 1.49 (95% confidence interval, 95% CI 1.04 to 1.94) lower zFEV1 and 0.84 (95% CI 0.44–1.23) lower zFEV1/FVC compared with controls. Between non-BPD-VLBW and controls we found significantly lower values also in all outcomes except zFVC. However, the differences with controls were clearly more pronounced among BPD-VLBW group. With a further adjustment for parental educational attainment, the differences remained largely similar (Supplementary Table S2). Subgroup analysis within the VLBW participants with or without BPD showed lower lung function among those with history of BPD and are presented in Supplementary Table S3. We conducted an additional analyses in which BPD-VLBW and non-BPD-VLBW groups were defined by the need for supplemental oxygen for more than 28 days or for more than 36 weeks (Supplementary Table S4). When BPD was defined by the need for supplemental oxygen >28 days, BPD-VLBW participants had 1.23 (95% CI 0.85–1.62) lower zFEV1 and 0.79 (95% CI 0.44–1.14) lower zFEV1/FVC, compared with term-born controls. These differences were smaller than those in our primary analysis where BPD was defined by a clinician’s diagnosis. The corresponding numbers for BPD definition by the need for supplemental oxygen for more than 36 weeks were for zFEV1 1.97 (95% CI 1.31–2.62) and for zFEV1/FVC 1.28 (95% CI 0.7–1.83). These differences were larger than those where BPD was defined by a clinician’s diagnosis.

### Respiratory symptoms and use of medication in mid-adulthood

We then assessed respiratory symptoms assessed by self-report questionnaire. Asthma diagnosed by a physician was reported by 31 (27.0%) VLBW, 4 (20.0%) BPD-VLBW, 27 (28.4%) non-BPD-VLBW) and 30 (21.1%) control participants (Supplementary Table S5). From these participants, 9 VLBW (7.8%) and 8 controls participants (5.6%) reported having the asthma diagnosis within the past 12 months from the clinical visit.

VLBW participants were more likely to report use of any obstructive airway disease medication (18.3% vs. 8.5%, odds ratio, OR 2.39, 95% CI 1.11–5.15) compared with term-born peers. In addition, VLBW participants were more likely to report an attack of asthma (7.1% vs. 1.4%, OR 4.95 95% CI 1.01–24.29) in the past 12 months. Otherwise, VLBW participants did not report more respiratory symptoms in the past 12 months (Supplementary Table S5).

### Lung function comparison from young to mid-adulthood

**Fig. 2:**
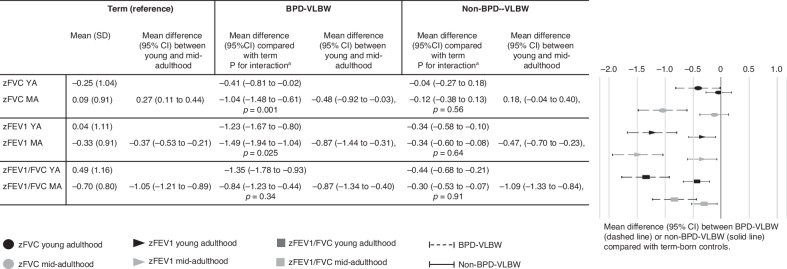
FVC, FEV1, and FEV1/FVC z-score mean differences (95% CI) from participants compared with controls in young and mid-adulthood, adjusted for group, age, sex and cohort. FVC forced vital capacity, FEV1 forced expiratory volume in 1s, FEV1/FVC ratio of the forced expiratory volume in the first 1s to the forced vital capacity, MA mid-adulthood, VLBW very low birth weight, SD standard deviation, YA young adulthood. aInteraction between group (BPD-VLBW or non-BPD-VLBW vs. control) * assessment timepoint (young vs. mid-adulthood).

The young adulthood lung function outcomes, which have been previously reported separately in each cohort,^7,23^ were now pooled together, reanalyzed by linear regression and described as a combined analysis including both cohorts in Table 2 and Fig. 2. We found differences in both groups; between BPD-VLBW compared with control participants and between non-BPD-VLBW and controls, in FVC, FEV1, FEV1/FVC, FEF_25–75%_, and FEF_75%_ absolute values and z-scores. Only zFVC and zFEF_75%_ did not reach statistical significance among non-BPD-VLBW group compared with controls.

We then assessed by mixed model whether these differences of BPD-VLBW and non-BPD-VLBW groups with controls are sustained in mid-adulthood. We found a significant age interaction in zFVC (*p* = 0.001) and zFEV1 (*p* = 0.025) between BPD-VLBW and control participants, indicating that BPD-VLBW participants have significantly faster decline of these parameters compared with their term-born peers. This is depicted in Fig. 2. Parallel to a stronger decline in zFVC compared with zFEV1, zFEV1/FVC increased between young and mid-adulthood in the BPD-VLBW group.

### Maternal smoking during pregnancy and smoking of the participant

Maternal smoking information was available only for HeSVA participants. In analysis model 3 (Supplementary Table S6), additional adjustment for maternal smoking during pregnancy had minimal effect on the results.

Of the 257 participants who were included in the spirometry analysis in mid-adulthood, 255 (99.2%) had reported their smoking status. None among the BPD-VLBW participants indicated to be smoking daily. In the non-BPD-VLBW group, 13 (11.5%) participants and in the control group 15 (10.6%) participants reported daily smoking. 29 (25.5%) VLBW participants and 42 (29.6%) controls reported that they have quit smoking at some point and 65 (57.5%) VLBW and 71 (50.0%) control participants indicated that they have never smoked.

Lung function results of VLBW and control participants who reported their smoking status in mid-adulthood and had spirometry values from young and mid-adulthood (*n* = 209) are reported in the Supplementary Table S7. VLBW participants with any kind of smoking history (daily/weekly/occasionally/quit at some point) had significantly lower FVC and FEV1 absolute values in both assessment points compared with controls who had a smoking history. In a time interaction analysis we found that rate of decline in FEV1/FVC (*p* = 0.003) and zFEV1/FVC (*p* = 0.009) was faster among VLBW participants who had any kind of smoking history, compared with control participants with any kind of smoking history (Supplementary Table S7).

### Non-participants

Our recently published detailed analysis of non-participants did not show any differences in neonatal characteristics between the participants who took part in the current mid-adulthood study and those who were invited but did not attend.^30^ In addition, we assessed whether participation rates differ between the BPD-VLBW, non-BPD-VLBW and control groups in HeSVA or NTNU LBW Life. Young adulthood data included altogether 32 VLBW participants with BPD (HeSVA *n* = 29, NTNU *n* = 3) diagnosed by a clinician, and mid-adulthood data 20 BPD-VLBW participants (HeSVA *n* = 16, NTNU *n* = 4). There was no difference in the participation rates between the BPD-VLBW, non-BPD-VLBW and control groups in HeSVA or NTNU at either time point (*Χ*^2^ *p* > 0.08). In addition, we conducted non-participant analyses among individuals who completed spirometry in young adulthood but did not attend mid-adulthood spirometry (Supplementary Table S8). These analyses showed no differences in neonatal and other background characteristics, except that younger VLBW participants (21.2, SD 2.6 years vs 21.9, SD 2.4 years, *p* = 0.04) who completed spirometry in young adulthood more likely attended the mid-adulthood spirometry. In addition, control participants who did not attend mid-adulthood spirometry had higher values in FEV1/FVC absolute values (90.13, SD 6.24 vs. 87.86, SD 7.46, *p* = 0.014) and in zFEV1/FVC (0.69, SD 1.09 vs. 0.32, SD 1.19, *p* = 0.018) in young adulthood (Supplementary Table S8).

## Discussion

### Key results

In mid-adulthood, VLBW participants, with or without BPD, had poorer lung function than their term-born peers. These differences were more pronounced among VLBW participants with a history of BPD, who had ~1.5 SD lower zFEV1 than their peers born at term. Between young and mid-adulthood, BPD-VLBW participants had a steeper decline in zFVC and zFEV1 compared with controls, but otherwise, differences in lung function remained largely similar between young and mid-adulthood clinical visits. VLBW participants who reported a history of smoking had a faster decline in FEV1/FVC and zFEV1/FVC compared with control participants with a smoking history.

### Strengths and limitations

To increase power and add precision, the mid-adulthood assessments were conducted in two birth cohorts with harmonized data collection and management procedures, training and audits to ensure similar measurements.

As to limitations, in spirometry assessments, different brands of devices were used between cohorts and between young and mid-adulthood. In addition to spirometry measurement and a respiratory health questionnaire, our study did not include other measurements related to lung physiology that would have allowed for a broader assessment of the participants’ respiratory health.

Our study may not directly reflect more contemporary cohorts since perinatal and neonatal care has evolved extensively in recent decades in terms of medications and non-invasive ventilation treatment that have increased survival and altered the characteristic phenotype of BPD. In our cohort, participants were born in the pre-surfactant era. No participant received surfactant as a part of clinical routine; seven HeSVA participants received it as a part of a randomized trial,^7^ not allowing a meaningful subgroup analysis. Moreover, the “old BPD” phenotype of acute lung injury in part related to mechanical ventilation may be more substantial than in contemporary cohorts, where the “new BPD” phenotype of impaired lung development may play a more central role.^31^ Our findings are thus most directly relevant for preterm VLBW survivors born in the pre-surfactant era, who are now entering middle age. These survivors constitute about one percent of their age group in high-resource settings.

VLBW participants were also more likely to receive medication for obstructive airway disease. This is consistent with the impairments in lung function; it may also attenuate the differences in lung function and give a more conservative estimate of the differences in lung function.

Our diagnosis of BPD was based on a clinician’s diagnosis. We chose this because of its use in previous papers^7,25^ and because in HeSVA it was confirmed carefully by a single neonatologist, based on Northway criteria,^24^ and in NTNU was available from neonatal medical records. We conducted sensitivity analyses: with a BPD criterion of supplementary oxygen at >28 days, the differences between VLBW and controls were smaller, and with a criterion of supplementary oxygen at 36 postmenstrual weeks, they were larger than on our primary analyses. We believe this indicates the robustness of our findings.

### Interpretation and consistency with previous research

Several studies have already indicated that VLBW adults have poorer respiratory health compared with their term-born peers with or without BPD.^6,7,32,33^

Many of these studies were included in an Adults Born Preterm International Collaboration individual participant data meta-analysis of nine cohorts^6^ that studied expiratory airflow from late adolescence to early adulthood among individuals born with VLBW or very preterm (VP). The results showed that VLBW and VP individuals’ airway capacity does not reach the normal peak compared with term-born peers, estimated by the measures of zFEV1, zFVC, zFEV1/FVC and zFEF_25–75%_ at mean age of 22 years. As to our knowledge, our study is first to report that VLBW adults with history of BPD would have a faster decline in zFVC and zFEV1 between 21 and 36 years of age.

Studies in children and adults born VP/VLBW have suggested a pattern of dysanapsis, a mismatch between a large lung volume and small airway caliber, which may result in obstructed airflow.^34^ Studies extending over the 30 s have however not confirmed this. Bårdsen et al.^19^ have studied lung function trajectories from childhood up to 35 years of age including age-related physiological decline (from 25 to 35 years) among individuals born extremely preterm (<28 weeks) or with extremely low birth weight (<1000 g) with or without BPD (defined by supplemental oxygen or respiratory support at postmenstrual age ≥36 weeks) and controls born at term. That study combined three cohorts, including individuals born during 1982–1985, 1991–1992 and 1999–2000. Cross-sectionally, the mid-adulthood assessment was largely similar to ours: reduced zFVC, proportionally more reduced zFVC resulting in a reduced zFEV1/FVC, not following a dysanapsis pattern. In contrast to us, they did not find a faster decline in FEV1 between the preterm-born group and controls.

The findings of our study are mostly in line with previous literature and adds to that by reporting for the first time a faster zFVC and zFEV1 rate of decline in the BPD-VLBW population. This suggests that BPD-VLBW adults’ vital capacity may decline faster compared with term-born peers. However, this was not reflected in self-reported respiratory symptoms, which were similar between groups.

In addition, our findings showed a steeper decline in zFEV1/FVC in VLBW participants with smoking history compared with controls with smoking history, suggesting that VLBW airways may be particularly vulnerable to smoking.^35^

Lifetime trajectories of FEV1, FEV1/FVC, FVC and their combinations have been assessed in several studies.^6,19,36,37^ A prime example is the Tasmanian Longitudinal Health Study^38^ that estimated concurrently both obstructive and restrictive lifetime patterns from childhood (7 years) to 53 years of age. It found that at the age of 53, individuals who had a low FVC trajectory (restrictive-only pattern) but not FEV1 or FEV1/FVC had evidence of true lung restriction and were characterized by risk factors and comorbidities such as childhood underweight, adult obesity, diabetes, cardiovascular conditions, hypertension and obstructive sleep apnea.^37^ At the same age, the study has observed an increased risk of COPD (OR 2.9, 95% CI 1.1–7.7) among those born very to moderate preterm (gestational age 28–33 completed weeks).^39^

As to clinical implications, clinicians who see adults born preterm with VLBW should be vigilant for respiratory symptoms. In all adults who present with respiratory symptoms, we argue that a full medical history should routinely include key birth characteristics such as preterm birth.

In conclusion, adults born preterm with VLBW have poorer lung function compared with their term-born peers. The differences are more pronounced among VLBW individuals with a history of BPD, who have up to 1.5 SD lower zFEV1 than their peers born at term. The differences remain largely similar between young and mid-adulthood. Among BPD-VLBW participants, we found faster decline of zFVC and zFEV1, which may indicate a more rapid downward trajectory of functional lung volume and a higher risk of respiratory disease later in life. A comprehensive medical history for adults presenting with respiratory symptoms should include details of birth characteristics, especially whether they were born preterm or had a history of BPD.

## Supplementary information

supplemental tables

supplemental table S8

## Data Availability

The datasets generated and/or analyzed during the current study include sensitive health data and cannot be made publicly available. Aggregated data are available from the corresponding author for reasonable requests.
