# Supplementary material for: Lung function in adults born with very low birth weight from young to mid-adulthood
Source: Pediatr Res. 2025 Jun 28;99(1):215–23. doi: 10.1038/s41390-025-04246-z (PMC12920113; doi:10.1038/s41390-025-04246-z)
Supplement: Supplementary file 1 — supplemental tables [file 41390_2025_4246_MOESM1_ESM.pdf]

Supplemental table S1. Comparisons of lung function from young to mid-adulthood between VLBW and term-born control participants

	**All VLBW**	**Controls (reference)**		
Young adulthood (n)	198	225		
Age (years)	21.6 (2.5)	21.4 (2.6)		
Mid-adulthood study (n)	115	142		
Age (years)	36.1 (3.3)	35.8 (3.3)		
**Absolute values**	**Mean (SD)**	**Mean (SD)**	**Mean difference (95%CI)[a]**	**p-value for age interaction[b]**
FVC young adulthood (l)	4.27 (1.03)	4.62 (1.01)	**-0.37 (-0.50 to -0.23)**	
FVC mid-adulthood study (l)	4.23 (1.11)	4.76 (1.04)	**-0.46 (-0.62 to -0.30)**	0.13
FEV1 young adulthood (l)	3.63 (0.83)	4.08 (0.85)	**-0.49 (-0.60 to -0.37)**	
FEV1 mid-adulthood study (l)	3.16 (0.82)	3.68 (0.76)	**-0.47 (-0.60 to -0.33)**	0.59
FEV1FVC young adulthood (%)	85.82 (9.23)	88.97 (7.0)	**-3.68 (-5.16 to -2.20)**	
FEV1FVC mid-adulthood (%)	75.20 (7.91)	77.80 (5.51)	**-2.61 (-4.21 to 1.01)**	0.93
FEF 25-75% young adulthood[c]	4.16 (1.36)	4.85 (1.26)	**-0.70 (-0.96 to -0.43)**	
FEF 25%-75% mid-adulthood	2.68 (0.93)	3.32 (0.83)	**-0.60 (-0.80 to -0.40)**	0.86
FEF 75 % young adulthood[c]	2.37 (0.95)	2.80 (0.88)	**-0.44 (-0.63 to -0.24)**	
FEF 75 % mid-adulthood study	1.17 (0.52)	1.53 (0.50)	**-0.32 (-0.44 to -0.21)**	0.37
**z-scores**				
FVC young adulthood	-0.38 (1.19)	-0.25 (1.04)	-0.11 (-0.32 to 0.11)	
FVC mid-adulthood study	-0.25 (1.2)	0.09 (0.91)	**-0.28 (-0.52 to -0.03)**	0.11
FEV1 young adulthood	-0.42 (1.36)	0.04 (1.11)	**-0.49 (-0.73 to -0.26)**	
FEV1 mid-adulthood study	-0.92 (1.29)	-0.33 (0.91)	**-0.53 (-0.80 to -0.27)**	0.25
FEV1FVC young adulthood	0.004 (1.41)	0.49 (1.16)	**-0.59 (-0.82 to -0.36)**	
FEV1FEVC mid-adulthood study	-1.08 (1.05)	-0.70 (0.80)	**-0.39 (-0.61 to -0.17)**	0.76
FEF 25%-75% young adulthood[c]	-0.19 (1.35)	0.33 (1.10)	**-0.51 (-0.78 to -0.24)**	
FEF 25%-75% mid-adulthood study	-1.21 (1.09)	-0.63 (0.81)	**-0.57 (-0.80 to -0.34)**	0.34
FEF 75% young adulthood[c]	0.29 (1.18)	0.69 (0.97)	**-0.39 (-0.62 to -0.15)**	
FEF 75% mid-adulthood study	-0.74 (1.05)	-0.18 (0.76)	**-0.54 (-0.76 to -0.32)**	0.15

Abbreviations: CI, confidence interval; FVC, forced vital capacity; FEV1, forced expiratory volume in 1 second; FEV1/FVC, ratio of the forced expiratory volume in the first one second to the forced vital capacity; $FEF_{25-75\%}$, forced expiratory flow at 25% to 75%; $FEF_{75\%}$, forced expiratory flow when 75% of FVC has been exhaled; VLBW, Very low birth weight, SD: standard deviation

[a]Linear regressions adjusted for cohort, age and sex

[b]p-value for age interaction by mixed models

[c]data available only for Helsinki Study of Very Low Birth Weight adults (HeSVA) participants

Supplemental table S2. Additional adjustment models of mean differences in lung function in mid-adulthood in adults born with VLBW with or without BPD compared with term-born controls

**z-scores**	**Model**[a]	**1.BPD-VLBW**	**2.non-BPD-VLBW**	**3.Term (reference)**	**Group 1 compared with controls**	**Group 2 compared with controls**
		Mean (SD)	Mean (SD)	Mean (SD)	Mean difference (95%CI)[a]	Mean difference (95%CI)[a]
**FVC**	1	-1.10 (1.14)	-0.08 (1.15)	0.09 (0.91)	**-1.04 (-1.48 to -0.61)**	-0.12 (-0.38 to 0.13)
	2				**-1.13 (-1.58 to -0.68)**	-0.17 (-0.43 to 0.10)
**FEV1**	1	-1.94 (1.26)	-0.70 (1.19)	-0.33 (0.91)	**-1.49 (-1.94 to -1.04)**	**-0.34 (-0.60 to -0.08)**
	2				**-1.62 (-2.01 to -1.14)**	**-0.37 (-0.65 to -0.10)**
**FEV1/FVC**	1	-1.52 (1.03)	-0.99 (1.03)	-0.70 (0.80)	**-0.84 (-1.23 to -0.44)**	**-0.30 (-0.53 to -0.07)**
	2				**-0.90 (-1.31 to -0.50)**	**-0.30 (-0.53 to -0.06)**
**$FEF_{25-75\%}$**	1	-2.01 (1.28)	-1.05 (0.98)	-0.63 (0.81)	**-1.34 (-1.76 to -0.92)**	**-0.41 (-0.64 to -0.19)**
	2				**-1.44 (-1.88 to -1.00)**	**-0.43 (-0.66 to -0.20)**
**$FEF_{75\%}$**	1	-1.64 (1.22)	-0.55 (0.91)	-0.18 (0.76)	**-1.41 (-1.81 to -1.01)**	-0.22 (-0.46 to 0.02)
	2				**-1.46 (-1.88 to -1.05)**	**-0.36 (-0.58 to -0.15)**

Abbreviations: BPD, bronchopulmonary dysplasia; CI, confidence interval; FVC, forced vital capacity; FEV1, forced expiratory volume in 1 second; FEV1/FVC, ratio of the forced expiratory volume in the first one second to the forced vital capacity; $FEF_{25-75\%}$, forced expiratory flow at 25% to 75%; $FEF_{75\%}$, forced expiratory flow when 75% of FVC has been exhaled; VLBW, Very low birth weight; SD, standard deviation

[a]Linear regressions: model 1 adjusted for cohort, age and sex and model 2 adjusted for cohort, age, sex and parental educational attainment

Supplemental table S3. Lung function analysis within VLBW group with or without BPD in young and mid-adulthood adjusted for cohort, age and sex.

	1. BPD-VLBW	2. Non-BPD-VLBW		
Young adulthood n	32	166		
Age, years (SD)	21.4 (2.5)	21.6 (2.5)		
Mid-adulthood study n	20	95		
Age, years (SD)	36.3 (2.7)	36.1 (3.4)		
**Absolute values**	**Mean (SD)**	**Mean (SD)**	**Mean difference (CI95%)[a]**	**P-value for age interaction[b]**
FVC young adulthood (l)	3.86 (0.88)	4.34 (1.03)	**-0.41 (-0.69 to -0.12)**	
FVC mid-adulthood (l)	3.59 (1.09)	4.36 (1.07)	**-0.67 (-1.00 to -0.34)**	**0.01**
FEV1 young adulthood (l)	3.16 (0.86)	3.72 (0.80)	**-0.56 (-0.80 to -0.33)**	
FEV1 mid-adulthood (l)	2.58 (0.82)	3.28 (0.77)	**-0.63 (-0.91 to -0.35)**	0.14
FEV1/FVC young adulthood (%)	81.49 (10.77)	86.65 (8.76)	**-6.59 (-9.84 to -3.34)**	
FEV1/FEVC mid-adulthood (%)	71.95 (8.70)	75.89 (7.60)	**-3.95 (-7.72 to -0.18)**	0.55
**z-scores**				
FVC young adulthood	-0.75 (1.07)	-0.31 (1.20)	-0.45 (-0.91 to 0.007)	
FVC mid-adulthood	-1.10 (1.14)	-0.08 (1.15)	**-0.86 (-1.39 to -0.33)**	**0.01**
FEV1 young adulthood	-1.17 (1.36)	-0.27 (1.32)	**-1.03 (-1.52 to -0.53)**	
FEV1 mid-adulthood	-1.94 (1.26)	-0.70 (1.19)	**-1.10 (-1.69 to -0.52)**	0.12
FEV1/FVC young adulthood	-0.66 (1.48)	0.13 (1.37)	**-0.96 (-1.46 to -0.47)**	
FEV1/FEVC mid-adulthood	-1.51 (1.03)	-0.99 (1.03)	**-0.53 (-1.04 to -0.03)**	0.45

Abbreviations: BPD, bronchopulmonary dysplasia; CI, confidence interval; FVC, forced vital capacity; FEV1, forced expiratory volume in 1 second; FEV1/FVC, ratio of the forced expiratory volume in the first one second to the forced vital capacity; $FEF_{25-75\%}$, forced expiratory flow at 25% to 75%; $FEF_{75\%}$, forced expiratory flow when 75% of FVC has been exhaled; VLBW; Very low birth weight; SD, standard deviation

[a]Linear regressions adjusted for cohort, age and sex

[b]p-value for age interaction by mixed model analysis

Supplemental table S4. Spirometry result comparisons from mid-adulthood study between VLBW participants with BPD defined more than 28 days or 36 weeks of supplemental oxygen and term-born controls

**Variable**	**1.BPD-VLBW (oxygen at 28 days)** n=29	**2.BPD-VLBW (oxygen at 36 weeks)[a]** n=9	**3.Term (reference)** n=142	**Group 1 vs. term**	**Group 2 vs. term**
**Absolute values**	Mean (SD)	Mean (SD)	Mean (SD)	Mean difference (95%CI)[b]	Mean difference (95%CI)[b]
FVC (l)	3.94 (1.02)	3.62 (1.19)	4.76 (1.04)	**-0.75 (-0.99 to -0.50)**	**-0.97 (-1.38 to -0.56)**
FEV1 (l)	2.82 (0.73)	2.47 (0.92)	3.68 (0.76)	**-0.81 (-1.01 to -0.61)**	**-1.09 (-1.43 to -0.75)**
FEV1/FVC (%)	72.04 (9.41)	67.95 (10.05)	77.80 (5.51)	**-5.73 (-8.23 to -3.23)**	**-10.03 (-13.92 to -6.15)**
**z-score**					
FVC	-0.77 (1.07)	-1.24 (1.54)	0.09 (0.91)	**-0.74 (-1.11 to -0.37)**	**-1.23 (-1.90 to -0.65)**
FEV1	-1.66 (1.18)	-2.34 (1.72)	-0.33 (0.91)	**-1.23 (-1.62 to -0.85)**	**-1.97 (-2.62 to -1.31)**
FEV1/FVC	-1.47 (1.17)	-1.99 (1.04)	-0.70 (0.80)	**-0.79 (-1.14 to -0.44)**	**-1.28 (-1.83 to -0.74)**

Abbreviations: BPD, bronchopulmonary dysplasia; CI, confidence interval; FVC, forced vital capacity; FEV1, forced expiratory volume in 1 second; FEV1/FVC, ratio of the forced expiratory volume in the first one second to the forced vital capacity; VLBW, Very low birth weight; SD, standard deviation

[a]oxygen at 36 postmenstrual weeks

[b]Linear regression adjusted for cohort, age and sex

Supplemental table S5. Respiratory symptoms between VLBW participants with or without BPD and term-born controls in mid-adulthood.

	**VLBW**	**Term (reference)**	Odds ratio CI95%[c]	p-value
Number of participants	**115**	**142**		
History of asthma diagnosed by a physician	31 (27.0%)	30 (21.1%)	1.41 (0.70 to 2.52)	0.25
Use of any medication for obstructive airway disease[a]	**21 (18.3%)**	**12 (8.5%)**	**2.39 (1.11 to 5.15)**	**0.026**
Use of inhaled adrenergic bronchodilators[b]	17 (14.8%)	10 (7.0%)	2.26 (0.98 to 5.19)	0.055
**Respiratory symptoms in the past 12 months:**				
Wheezing without cold	19 (36.5%)	19 (30.6%)	1.28 (0.58 to 2.82)	0.54
with medication[a]	15 (83.3%)	6 (60.0%)	4.54 (0.49 to 42.44)	0.18
without medication[a]	4 (11.8%)	13 (25.0%)	0.35 (0.10 to 1.25)	0.11
Woken up with a feeling of tightness in the chest	15 (17.6%)	15 (15.3%)	1.20 (0.54 to 2.66)	0.66
with medication[a]	9 (47.4%)	4 (36.4%)	1.22 (0.20 to 7.39)	0.83
without medication[a]	6 (9.1%)	11 (12.6%)	0.72 (0.24 to 2.15)	0.56
Woken up by an attack of shortness of breath	8 (7.1%)	9 (6.4%)	1.15 (0.42 to 3.14)	0.78
with medication[a]	3 (14.3%)	2 (16.7%)	0.68 (0.08 to 6.08)	0.68
without medication[a]	5 (5.4%)	7 (5.4%)	1.13 (0.34 to 3.84)	0.84
Woken up by an attack of coughing	28 (24.8%)	26 (18.4%)	1.44 (0.77 to 2.68)	0.25
with medication[a]	10 (47.6%)	4 (33.3%)	1.27 (0.25 to 6.54)	0.77
without medication[a]	18 (19.6%)	22 (17.1%)	1.21 (0.59 to 2.48)	0.61
Attack of asthma	**8 (7.1%)**	**2 (1.4%)**	**4.95 (1.01 to 24.29)**	**0.049**
with medication[a]	8 (38.1%)	2 (16.7%)	3.46 (0.47 to 25.62)	0.22
without medication[a]	-	-	-	-

Abbreviations: CI, confidence interval; VLBW, Very low birth weight

[a]including medication with ATC code R03AC or R03AK or R03BA or R03DC

[b]including medication with ATC R03AC, participant may have used (in addition other respiratory medication)

[c]odds ratio by logistic regression adjusted for cohort, age and sex

Supplemental table S6. Additional adjustment models of mean differences in lung function in mid-adulthood in adults born with VLBW with or without BPD compared to term-born controls from HeSVA participants

		1.BPD-VLBW	2.non-BPD-VLBW	3.Term (reference)	BPD-VLBW vs. Term	non-BPD-VLBW vs. Term
**n**		16	61	83		
**z-score**	**Model***	Mean (SD)	Mean (SD)	Mean (SD)	Mean difference (95%CI)[a]	Mean difference (95%CI)[a]
**FVC**	1	-1.11 (0.93)	-0.32 (1.11)	-0.19 (0.85)	**-0.91 (-1.39 to -0.44)**	-0.13 (-0.45 to 0.20)
	2				**-0.98 (-1.48 to -0.49)**	-0.14 (-0.46 to 0.19)
	3				**-1.03 (-1.61 to -0.45)**	-0.01 (-0.37 to 0.35)
**FEV1**	1	-1.83 (0.91)	0.91 (1.22)	-0.56 (0.91)	**-1.23 (-1.73 to -0.73)**	-0.35 (-0.71 to 0.00)
	2				**-1.32 (-1.84 to -0.80)**	**-0.37 (-0.72 to -0.10)**
	3				**-1.46 (-2.07 to -0.86)**	-0.18 (-0.59 to 0.24)
**FEV1/FVC**	1	1.32 (0.91)	-0.99 (1.00)	-0.65 (0.82)	**-0.62 (-1.06 to -0.17)**	**-0.36 (-0.66 to -0.70)**
	2				**-0.67 (-1.14 to -0.20)**	**-0.38 (-0.67 to -0.08)**
	3				**-0.81 (-1.35 to -0.26)**	-0.26 (-0.60 to 0.08)
**$FEF_{25-75\%}$**	1	-1.78 (1.03)	-1.11 (0.96)	-0.66 (0.90)	**-1.04 (-1.54 to -0.54)**	**-0.46 (-0.76 to -0.15)**
	2				**-1.13 (-1.65 to -0.61)**	**-0.48 (-0.78 to -0.17)**
	3				**-1.22 (-1.81 to -0.63)**	-0.30 (-0.65 to 0.50)
**$FEF_{75\%}$**	1	-1.33 (0.87)	-0.63 (0.80)	-0.29 (0.75)	**-0.98 (-1.39 to -0.57)**	**-0.36 (-0.61 to -0.10)**
	2				**-1.02 (-1.45 to -0.58)**	**-0.37 (-0.63 to -0.12)**
	3				**-1.15 (-1.66 to -0.64)**	-0.20 (-0.50 to 0.09)

Abbreviations: BPD, bronchopulmonary dysplasia; CI, confidence interval; FVC, forced vital capacity; FEV1, forced expiratory volume in 1 second; FEV1/FVC, ratio of the forced expiratory volume in the first one second to the forced vital capacity; $FEF_{25-75\%}$, forced expiratory flow at 25% to 75%; $FEF_{75\%}$, forced expiratory flow when 75% of FVC has been exhaled; VLBW, Very low birth weight; SD, standard deviation

[a]Linear regressions: model 1 adjusted for cohort, age and sex, model 2 adjusted for cohort, sex and parental educational attainment and model 3 adjusted for cohort, age, sex, parental educational attainment and maternal smoking

Supplemental table S7. Lung function among those who according to mid-adulthood assessment questionnaire indicated any kind of smoking history with those who have never smoked and have spirometry results from young and mid-adulthood assessments.

	**1. VLBW, never smoked**	**2. VLBW with smoking history**	**3. Control, never smoked**	**4. Control with smoking history**	**1. vs. 2. mean difference (CI95%)[a]**	**3. vs. 4. mean difference (CI95%)[a]**	**2. vs. 4. mean difference (CI95%)[a]**	**1. vs. 2. Age int.[b]**	**3. vs. 4. Age int.[b]**	**2. vs. 4. Age int.[b]**
N	55	41	53	60						
**Absolute values**	Mean (SD)	Mean (SD)	Mean (SD)	Mean (SD)						
FVC YA[a] (l)	4.26 (1.05)	4.21 (1.07)	4.44 (0.92)	4.91 (1.08)	-0.12 (-0.42 to 0.18)	-0.13 (-0.38 to 0.12)	**-0.38 (-0.66 to -0.10)**			
FVC MA[a] (l)	4.19 (1.07)	4.19 (1.16)	4.67 (0.91)	4.86 (1.20)	-0.20 (-0.50 to 0.10)	0.06 (-0.17 to 0.29)	**-0.31 (-0.59 to -0.03)**	0.73	**0.010**	0.83
FEV1 YA (l)	3.53 (0.83)	3.69 (0.86)	3.94 (0.79)	4.22 (0.88)	**-0.28 (-0.53 to -0.02)**	-0.01 (-0.24 to 0.21)	**-0.31 (-0.57 to -0.05)**			
FEV1 MA (l)	3.11 (0.81)	3.15 (0.87)	3.62 (0.69)	3.73 (0.85)	-0.18 (-0.44 to 0.09)	0.06 (-0.12 to 0.24)	**-0.35 (-0.58 to -0.12**)	0.27	**0.045**	0.68
FEV1/FVC YA (%)	83.62 (9.80)	88.49 (7.90)	89.12 (7.39)	86.62 (7.43)	**-4.38 (-7.87 to -0.90)**	1.90 (-0.85 to 4.66)	0.49 (-2.46 to 3.44)			
FEV1/FVC MA (%)	74.40 (8.37)	75.78 (7.70)	77.94 (4.29)	77.43 (6.46)	-0.99 (-4.32 to 2.33)	-0.03 (-2.13 to 2.06)	-2.57 (-5.28 to 0.14)	**0.021**	0.052	**0.003**
**z-scores**										
FVC YA	-0.51 (1.10)	-0.18 (1.30)	-0.27 (0.89)	-0.05 (1.01)	-0.36 (-0.84 to 0.13)	-0.24 (-0.60 to 0.12)	-0.07 (-0.54 to 0.41)			
FVC MA	-0.52 (1.08)	-0.11 (1.16)	0.22 (0.92)	-0.01 (0.93)	**-0.48 (-0.91 to -0.05)**	0.14 (-0.20 to 0.47)	0.04 (0.37 to 0.44)	0.74	**0.008**	0.92
FEV1 YA	-0.77 (1.21)	0.01 (1.41)	-0.004(1.01)	0.07 (1.15)	**-0.77 (-1.30 to -0.23)**	-0.07 (-0.48 to 0.35)	-0.07 (-0.60 to 0.46)			
FEV1 MA	-1.20 (1.23)	-0.75 (1.22)	-0.21 (0.89)	-0.43 (0.92)	-0.48 (-0.98 to 0.01)	0.12 (-0.22 to 0.46)	-0.26 (-0.68 to 0.16)	0.17	0.09	0.25
FEV1/FVC YA	-0.34 (1.43)	0.34 (1.21)	0.46 (1.27)	0.18 (1.11)	**-0.63 (-1.15 to -0.10)**	0.33 (-0.12 to 0.77)	0.03 (-0.44 to 0.50)			
FEV1/FVC MA	-1.15 (1.09)	-1.00 (1.03)	-0.70 (0.66)	-0.68 (0.92)	-0.10 (-0.55 to 0.34)	-0.02 (-0.33 to 0.29)	**-0.41 (-0.80 to -0.02)**	**0.02**	0.073	**0.009**

Abbreviations: CI, confidence interval; FVC, forced vital capacity; FEV1, forced expiratory volume in 1 second; FEV1/FVC, ratio of the forced expiratory volume in the first one second to the forced vital capacity; MA, Mid-adulthood assessment; SD, standard deviation; VLBW, Very low birth weight; YA, Young adulthood assessment

[a]Linear regressions adjusted for cohort, age and sex

[b]p-value for age interaction by mixed models
